# Effects of long-term Tai-Chi Chuan practice on whole-body balance control during obstacle-crossing in the elderly

**DOI:** 10.1038/s41598-022-06631-8

**Published:** 2022-02-17

**Authors:** Chien-Chung Kuo, Sheng-Chang Chen, Tsan-Yang Chen, Tsung-Jung Ho, Jaung-Geng Lin, Tung-Wu Lu

**Affiliations:** 1 Department of Orthopedics, China Medical University Hospital, Taichung, Taiwan, ROC; 2 Department of Orthopedics, School of Medicine, China Medical University, Taichung, Taiwan, ROC; 3 Department of Biomedical Engineering, National Taiwan University, Taipei, Taiwan, ROC; 4 Integration Center of Traditional Chinese and Modern Medicine, Buddhist Tzu Chi General Hospital, Hualien City, Taiwan, ROC; 5 Department of Chinese Medicine, Buddhist Tzu Chi Hospital, Hualien City, Taiwan, ROC; 6 School of Post-Baccalaureate Chinese Medicine, Tzu Chi University, Hualien City, Taiwan, ROC; 7 Institute of Chinese Medical Science, China Medical University, Taichung, Taiwan, ROC

**Keywords:** Biomedical engineering, Applied physics, Lifestyle modification

## Abstract

Older people are subject to an increased risk of falling compared to the young, especially during obstacle negotiation. This study aimed to quantify the effects of long-term Tai-Chi Chuan (TCC) practice on the balance control during obstacle-crossing in older people in terms of the inclination angles (IA) of the body’s centre of mass (COM) relative to the centre of pressure (COP), and the rate of change of IA (RCIA). Fifteen healthy older adults who had practised TCC for at least 13 years and 15 healthy controls without any experience in TCC performed obstacle-crossing in a gait laboratory. The TCC group showed significantly greater leading and trailing toe-obstacle clearances but smaller trailing stride lengths when compared to controls. In the sagittal plane, the TCC group showed significantly smaller average anterior IA when the COM was anterior to the COP but greater average posterior IA when the COM was posterior to the COP, with significantly smaller average and peak RCIA over the crossing cycle. Long-term TCC practitioners showed an obstacle-crossing technique for less risk of tripping and better balance control, as indicated respectively by significantly increased toe-obstacle clearances and more posterior COM position relative to the COP with smaller anterior IA and RCIA during leading crossing and greater posterior IA and frontal RCIA at trailing-toe crossing. These benefits appeared to be related to the main features of TCC movements that emphasized maintaining balance during single-leg support and keeping the body weight on the trailing limb during the slow weight-shifting of double-limb support.

## Introduction

Tripping over obstacles is considered one of the most common causes of falls in older people^1–3^. The older adults were found to increase the foot-clearance^4–6^ and increased lead foot-clearance linearly with increasing obstacle height^9^, apart from slower crossing speed, and shorter step length and heel-obstacle distance compared with young adults^4–8^. Increasing foot-clearance to avoid tripping^4,9,10^ may lead to perturbations to balance and whole-body posture changes, which is, in turn, another major contributing factor to falls in older people^1,2,11^. Alterations in whole-body posture may include smaller frontal pelvic motion^12^, greater variabilities in the lower limb inter-joint coordination^13^, and altered COM-COP control^14^, leading to a greater risk of imbalance during obstacle-crossing. Once a trip or loss of balance occurs, failure to recover balance would lead to falls. Therefore, understanding the changes in the balance control of older people is essential in developing and evaluating methods for reducing fall risks.

The body’s balance control during locomotion has been described by the motion of the body’s COM relative to the COP in various populations during dynamic activities^5,15–17^. Both the COM-COP inclination angle (IA), namely the angle formed by the COM-COP vector and the vertical vector, and the rate of change of IA (RCIA), i.e., the change of IA over a given short time interval, are sensitive indices for quantifying the actual COM-COP control^18,19^. Generally, the greater the IA, the greater the COM-COP vector deviates from the vertical, and the greater the effort (e.g., joint torque) needed to reduce or maintain the IA unless accompanied by an appropriate RCIA for dynamic balance, corresponding to the position and velocity control of the COM described by Pai and Patton^19,20^. Increased toe-obstacle clearance during obstacle crossing demonstrated by older people reduces the risk of tripping^4,6,9,10,13^. However, the COM-COP control becomes more challenging, requiring extra joint torques to maintain the body’s dynamic stability unless accompanied by an increased RCIA. To compensate for the more difficult COM-COP control when obstacle height was increased, healthy older people tended to reduce the separation between COM and COP in order to reduce the mechanical load on the supporting limb^5,14^. Despite this control strategy however, older people still displayed greater IAs with smaller RCIAs in the sagittal plane at leading-toe crossing and demonstrated greater RCIA for the same IA as young controls during the rest of the crossing cycle^5^. These results suggest that ageing may compromise the ability of whole-body balance control described by the IA and RCIA in older people during obstacle-crossing. Therefore, balance training for safer obstacle-crossing may be needed for older people.

Although many training programmes addressing single factors, such as resistance, endurance, and feedback training, are available to older people to improve their balance capacity and reduce the risk of falling^21,22^, multi-factor exercises appear to be more effective in improving balance capability^23,24^. These multi-factor exercises can be structured to emphasize muscular strength/endurance training and other functions such as balance/somatosensory reaction and confidence. Tai-Chi Chuan (TCC), an ancient Chinese martial art, has been shown to improve multiple factors in older people^22,25–31^, and is a popular exercise among older people for enhancing their general physical condition. The practice of TCC consists of a series of slow, continuous, gentle motions transitioning from double-limb to single-limb support, thus focusing on dynamic weight shift to a narrower base of support. Participating in long-term TCC exercise can increase muscle strength^26,29,30^, flexibility^31,32^, balance^22,28,29^, sensory organization in postural control^27^, and reduced COP sway area during challenging tasks such as single-leg stance and tandem stance^25^. These changes are all critical components for preventing falls in older people^33,34^. TCC may cause practitioners to modify their gait and movement behaviours, leading them to move more cautiously. These modifications may provide some explanation for the observed reductions in falls^35^.

Knowledge of the effects of TCC on the COM-COP motions during obstacle-crossing may help better understand the balance control strategy and plan programmes to prevent fall-related injuries in older people. However, previous studies on older people with TCC experience during obstacle-crossing have focused mainly on the gait modifications in terms of temporospatial parameters, ground reaction forces, foot pressure distribution and joint kinematics^36–38^. Older people with TCC experience showed significantly larger forward ground reaction forces to propel the body with greater hip flexion of leading limb^36^, faster crossing velocity with altered pressure distribution in the trailing foot^37^, and longer periods of single-limb support^38^ while crossing obstacles. No study has reported the effects of TCC on foot-clearances and the whole-body balance control in terms of IA and RCIA in older people when crossing obstacles of different heights. To form a complete assessment of the TCC effects on balance control relevant to fall risks, data on the COM-COP motions in terms of IA and RCIA from both TCC and non-TCC practitioners are needed.

The purpose of the current study was to quantify the effects of long-term TCC practice and obstacle height on foot-clearances and the whole-body balance control in terms of IA and RCIA during obstacle-crossing in healthy older people. It was hypothesized that healthy older people with long-term experience in practising TCC would show more conservative COM-COP control with greater toe-obstacle clearance than those who did not practise TCC and that these results were not affected by obstacle heights.

## Materials and methods

All experiments of the current study were conducted with the approval of China Medical University Hospital Institutional Review Board (IRB No. DMR98-IRB-072). All the experiments and procedures conformed to the Ethical Principles for Medical Research Involving Human Subjects (World Medical Association Declaration of Helsinki). Fifteen healthy older adults who practised TCC at least 60 min a day and five days a week for thirteen or more years (TCC group, sex: 3 females/12 males, age: 72 ± 5.3 years, height: 162 ± 6.7 cm, mass: 58.3 ± 6.5 kg, TCC experience: 23 ± 10.5 years), and fifteen healthy controls who did leisure exercises (walking or jogging) for the same period, and matched with the TCC group for sex, age and BMI (Control group, sex: 3 females/12 males, age: 72 ± 6.0 years, height: 160 ± 5.7 cm, mass: 58 ± 10.4 kg) participated in the current study with informed written consent. They were all free of neuromusculoskeletal dysfunction and with normal or corrected vision. An a priori power analysis based on pilot results from four subjects for each group using G*POWER^39^ for two-way mixed-design analysis of variance (ANOVA) with one between-subject factor (group) and one within-subject factor (obstacle height) for the comparison of IA and RCIA determined that a projected sample size of 14 subjects for each group would be needed with a power of 0.8 and a medium to large effect size (Cohen's d > 0.5) at a significance level of 0.05. Thus, 15 subjects for each group were considered adequate for the main objectives of the current study.

In a hospital-based gait laboratory, each subject walked on a 10-m walkway at his/her preferred walking speed and crossed an obstacle made of an aluminium tube placed horizontally across a height-adjustable metal frame. The position and height of the obstacle placed in the middle of the walkway were defined by two infrared-retroreflective markers placed on each end of the tube. The poses of the body segments were tracked using 39 infrared-retroreflective markers placed on specific bony landmarks^18^. Three-dimensional marker trajectories were measured using a seven-camera motion capture system (Vicon 512, Oxford Metrics Group, UK) at 120 Hz, and the ground reaction forces (GRF) and the centre of pressure (COP) were measured at 1080 Hz using two forceplates (AMTI, USA) placed on either side of the obstacle. The test conditions included crossing obstacles of 10%, 20%, and 30% of the subject’s leg length—defined as the distance between the ipsilateral ASIS and medial malleolus—in a random order, with each lower limb leading^40^. The greatest obstacle height of 30% leg length was selected to cover the range of obstacles one might normally encounter in the daily environment because obstacles higher than 30% leg length are rare in daily activities^9,10,41^. Three obstacle heights enabled the trend of height effects to be determined^5,40–44^. A trial was defined as unsuccessful if the subject hit the obstacle during crossing. In the current study, all the subjects were able to cross the obstacles successfully without hitting the obstacle. Data for three complete crossing trials, each lasting about 30 s, for each lower limb leading were obtained for each obstacle height for each subject. Therefore, a total of 25–30 min was needed for the obstacle-crossing experiment for each subject. A 5-min break was allowed when changing obstacle-height conditions.

The body’s COM position was calculated as the mass-weighted sum of the positions of the COMs of all the body segments using the marker data and segmental inertial properties, which were obtained using a model-based optimization method that minimizes errors between model-predicted and measured COP positions during a number of calibration postures^45^. The motions of the COM relative to the COP were described using the inclination angles (IA) in the sagittal and frontal planes as follows:

1 $${\varvec{t}} = \left({\varvec{Z}} \times \frac{{{\varvec{P}}}_{COM-COP}}{\left|{{\varvec{P}}}_{COM-COP}\right|}\right),$$

2 $$\mathrm{Sagittal \; IA }= {\mathrm{sin}}^{-1}({t}_{Y}),$$

3 $$\mathrm{Frontal \; IA }=\left\{\begin{array}{l}-{\mathrm{sin}}^{-1}\left({t}_{X}\right), for \; the \; right \; limb\\ {\mathrm{sin}}^{-1}\left({t}_{X}\right), for \; the \; left \; limb,\end{array}\right.$$

where ***P***_*COM-COP*_ was the vector pointing from the COP to the COM in the laboratory coordinate system (**X**, **Y**, **Z**) with **X** pointing in the direction of progression. The RCIA was calculated by differentiating the time trajectories of IA using the GCVSPL method^46^. The leading limb was taken as the reference limb; thus, positive sagittal and frontal IA indicate a COM position anterior to the COP and away from the COP towards the trailing limb, respectively.

**Figure 1:**
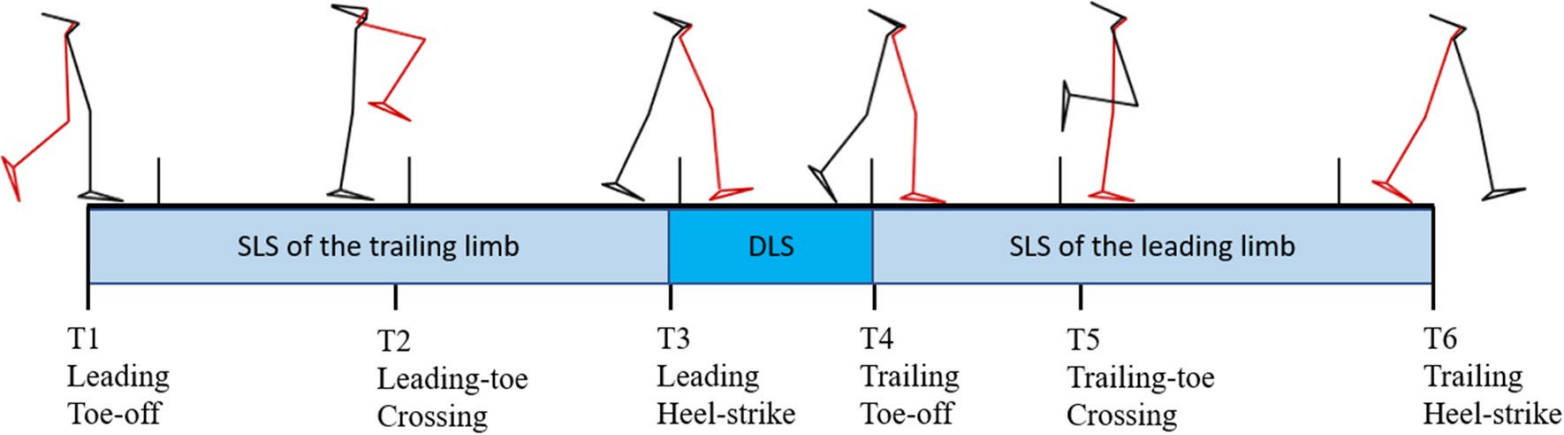
Schematic diagrams of the leading (red lines) and trailing (black lines) limbs at each event of the crossing cycle: leading toe-off (T1); leading toe above the obstacle (T2); leading heel-strike (T3); trailing toe-off (T4); trailing toe above the obstacle (T5); trailing heel-strike (T6).

The GRF and marker data were used to determine the gait events for the definition of a crossing cycle beginning at the toe-off of the leading limb before crossing (T1) followed by the heel-strike of the leading limb after crossing (T3) and toe-off of the trailing limb before crossing (T4), and ending at the heel-strike of the trailing limb after crossing (T6), as well as two critical events of crossing when the leading toe was above the obstacle (T2), and when the trailing toe was above the obstacle (T5) (Fig. 1). The marker data were also used to calculate end-point variables. The leading toe-obstacle clearance was calculated as the vertical distance of the toe marker above the obstacle at T2, while the trailing toe-obstacle clearance was the vertical distance of the toe marker above the obstacle at T5. The trailing toe-obstacle distance was defined as the horizontal distance between the obstacle and toe marker of the trailing limb at T2, and the leading heel-obstacle distance was defined as the horizontal distance between the obstacle and heel marker of the leading limb at T5. Crossing speed was also calculated as the average speed of the COM in the direction of progression over the crossing cycle. For subsequent statistical analysis, the values of the IA and RCIA when the leading and trailing toes were directly above the obstacle were obtained. The time-averaged values of IA and RCIA over the sub-phases of double-limb support (DLS, T3–T4) and single-limb support (SLS) of the leading (T1–T2 and T2–T3) and trailing (T4–T5) limb as well as the peak RCIA during DLS were also obtained for each test condition for each subject.

A two-way mixed-design analysis of variance (ANOVA) with one between-subject factor (group) and one within-subject factor (obstacle height) was performed to analyze the spatio-temporal parameters, namely crossing speed, stride length, toe-obstacle distance, heel-obstacle distance and toe-obstacle clearance. Previous studies have shown that crossing speed may affect the magnitudes of the IAs during locomotion^17,47^. Therefore, if there were no significant effects on crossing speed, each of the calculated IA and RCIA-related variables would be analyzed using the same two-way mixed-design ANOVA. If an interaction was found, pair-wise between-group comparisons would be performed using an independent *t*-test for each obstacle height, and a post-hoc trend analysis would be performed to determine the trend of the variable with increasing obstacle height for each group. If there was no interaction, the main effects would be reported. If a significant main height effect was found, a further polynomial test would be performed to determine the trend of the variable with increasing obstacle height. On the other hand, if there was a significant effect on crossing speed, a mixed-design two-way analysis of covariance (ANCOVA) with crossing speed as a covariate would be used instead for the analysis of the IA- and RCIA-related variables. All significance levels were set at α = 0.05. SPSS version 20 (SPSS Inc., Chicago, USA) was used for all statistical analyses.

## Results

There were no statistical interactions between group and obstacle height factors for any of the tested variables. Therefore, only the main effects are reported here. The TCC group showed significantly greater leading and trailing toe-obstacle clearances but smaller trailing stride lengths when compared to the control group (Table 1). No significant between-group differences were found in crossing speed, toe-obstacle and heel-obstacle distances (Table 1). With increasing obstacle height, the leading toe-obstacle clearances were significantly increased linearly, but the heel-obstacle distances, leading stride lengths and crossing speeds were decreased linearly (Table 1). Since significant obstacle height effects were found on crossing speed, the following results on IA and RCIA are adjusted values for differences in crossing speed.

**Table 1:** Means (standard deviations, SD) of the crossing speed, stride length, and toe-obstacle and heel-obstacle distances in the TCC and Control groups when crossing obstacles of different heights.

	Obstacle height (%LL)			Effects
	10	20	30	
**Crossing speed (m/s)**				
TCC	0.70 (0.06)	0.66 (0.07)	0.62 (0.07)	*p*_h_ = 0.01↓
Control	0.80 (0.14)	0.70 (0.12)	0.64 (0.11)	*p*_g_ = 0.17
**Leading stride length (% LL)**				
TCC	134.2 (8.0)	133.8 (8.4)	132.6 (8.3)	*p*_h_ = 0.01↓
Control	139.5 (11.7)	136.4 (11.1)	134.9 (12.1)	*p*_g_ = 0.33
**Trailing stride length (% LL)**				
TCC	128.0 (7.7)	128.6 (8.9)	129.2 (10.7)	*p*_h_ = 0.31
Control	141.7 (11.8)	139.1 (11.1)	137.3 (10.9)	*p*_g_ = 0.01*
**Toe-obstacle distance (% LL)**				
TCC	24.94 (4.8)	25.35 (4.8)	24.38 (5.1)	*p*_h_ = 0.28
Control	24.94 (4.4)	24.60 (4.2)	24.24 (4.8)	*p*_g_ = 0.86
**Heel-obstacle distance (% LL)**				
TCC	17.33 (2.8)	16.43 (3.3)	15.59 (3.5)	*p*_h_ = 0.01↓
Control	18.38 (5.1)	16.38 (3.7)	15.09 (4.5)	*p*_g_ = 0.89
**Leading-toe clearance (mm)**				
TCC	183.3 (23.4)	192.5 (24.5)	200.9 (19.1)	*p*_h_ = 0.01↑
Control	147.8 (47.8)	169.0 (32.9)	183.8 (42.8)	*p*_g_ = 0.04*
**Trailing-toe clearance (mm)**				
TCC	173.2 (31.1)	181.1 (41.3)	182.9 (45.7)	*p*_h_ = 0.33
Control	129.1 (45.6)	133.9 (51.9)	149.4 (55.3)	*p*_g_ = 0.01*

*LL* leg length, *p*_*h*_ *p*-value for obstacle height, *p*_*g*_ *p*-value for group

*Significant difference between subject groups

↑Significant linearly increasing trend

↓Significant linearly decreasing trend

**Figure 2:**
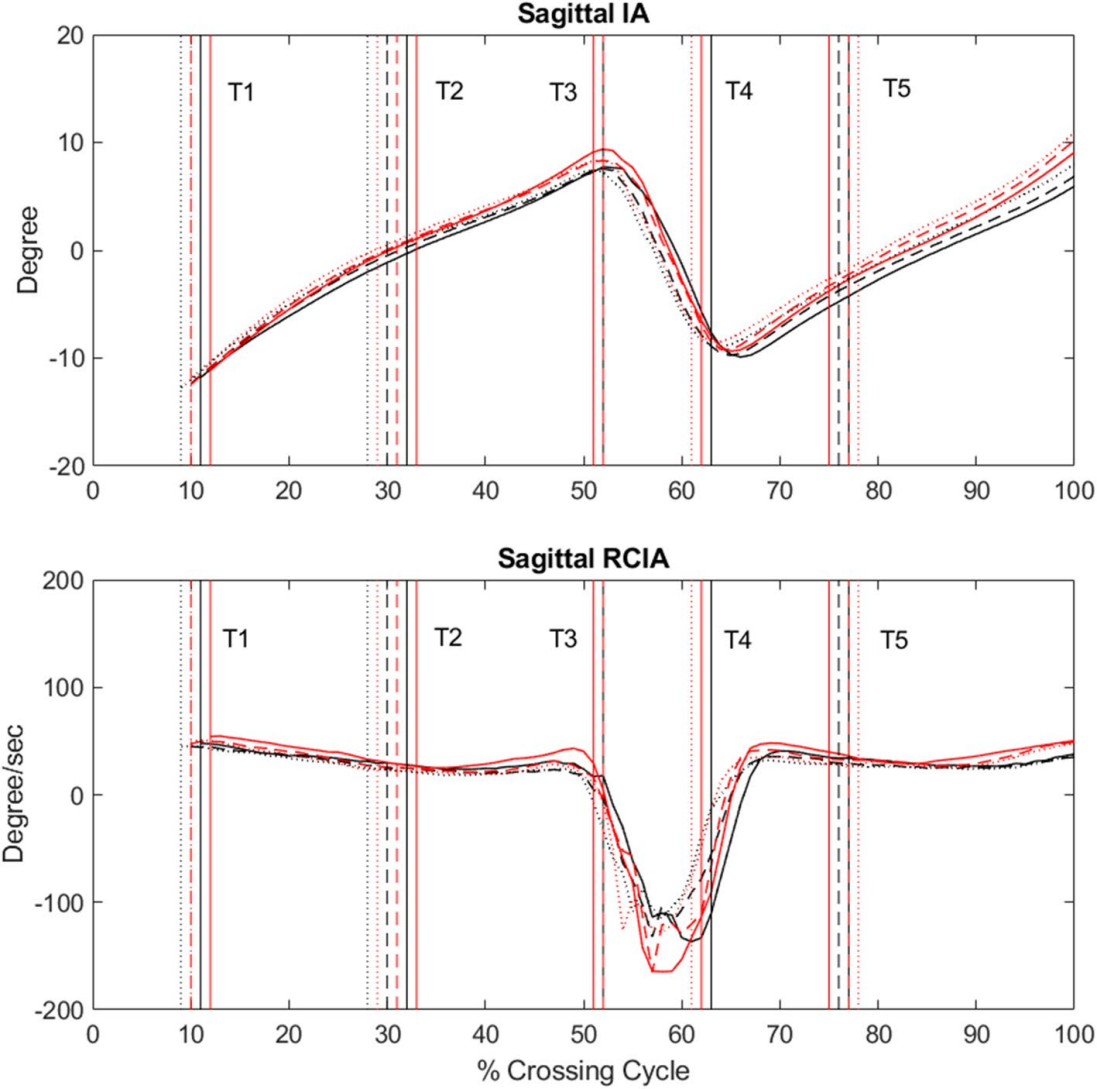
Ensemble-averaged sagittal IA and RCIA for the TCC (black curves) and Control (red curves) groups when crossing obstacles of 10% LL (solid), 20% LL (dashed), and 30% LL (dotted). Vertical lines indicate selected crossing events: leading toe-off (T1); leading toe above the obstacle (T2); leading heel-strike (T3); trailing toe-off (T4); trailing toe above the obstacle (T5).

In the sagittal plane, both Control and the TCC group showed similar patterns in the IA and RCIA curves but with significant differences in the magnitudes (Fig. 2). The TCC group showed significantly smaller average anterior IA during sub-phases when the COM was anterior to the COP (i.e., T2–T3 and T3–T4), but increased average posterior IA when the COM was posterior to the COP (i.e., T1–T2 and T4–T5), with significantly smaller magnitudes of average and peak RCIA over each sub-phase of the crossing cycle (Table 2). At the critical instances when the swing toe was above the obstacle, the TCC group also showed significantly smaller anterior IA at leading-toe crossing (T2) and greater posterior IA at trailing-toe crossing (T5) but with unaltered RCIA when compared to Control (Table 2). No significant height effects were found for any IA and RCIA variables in the sagittal plane (Table 2).

**Table 2:** Adjusted means (standard errors, SE) of the sagittal IA and RCIA at leading- and trailing-toe crossing, and of the average values over crossing sub-phases in the TCC and Control groups during obstacle-crossing. Statistical results using a mixed-model two-way analysis of covariance (ANCOVA) with crossing speed as a covariate are also shown. (T1: leading toe-off; T2: leading toe above the obstacle; T3: leading heel-strike; T4: trailing toe-off; T5: trailing toe above the obstacle).

	Group	Obstacle height (%LL)			Effects
		10	20	30	
**IA (degree)**					
T2	TCC	0.24 (0.26)	0.03 (0.27)	− 0.53 (0.26)	*p*_h_ = 0.81
	Control	1.73 (0.24)	1.00 (0.25)	0.78 (0.25)	*p*_g_ < 0.01*
T5	TCC	− 4.03 (0.24)	− 3.14 (0.28)	− 2.27 (0.36)	*p*_h_ = 0.14
	Control	− 2.83 (0.22)	− 1.8 (0.26)	− 0.95 (0.33)	*p*_g_ < 0.01*
T1–T2	TCC	− 4.97 (0.23)	− 5.26 (0.22)	− 5.63 (0.25)	*p*_h_ = 0.62
	Control	− 3.78 (0.21)	− 4.65 (0.21)	− 4.63 (0.23)	*p*_g_ = 0.01*
T2–T3	TCC	3.97 (0.21)	3.72 (0.18)	3.57 (0.22)	*p*_h_ = 0.83
	Control	5.11 (0.19)	4.73 (0.17)	4.51 (0.21)	*p*_g_ < 0.01*
T3–T4	TCC	0.65 (0.53)	0.08 (0.56)	− 0.17 (0.65)	*p*_h_ = 0.83
	Control	2.67 (0.53)	1.24 (0.56)	1.18 (0.65)	*p*_g_ = 0.05*
T4–T5	TCC	− 7.26 (0.22)	− 6.94 (0.18)	− 5.98 (0.23)	*p*_h_ = 0.42
	Control	− 6.78 (0.21)	− 5.82 (0.17)	− 5.01 (0.22)	*p*_g_ < 0.01*
**RCIA (degree/s)**					
T2	TCC	26.64 (1.40)	23.99 (1.43)	24.64 (1.25)	*p*_h_ = 0.99
	Control	25.34 (1.30)	24.59 (1.33)	21.16 (1.16)	*p*_g_ = 0.37
T5	TCC	32.61 (1.67)	30.05 (1.69)	27.44 (1.61)	*p*_h_ = 0.99
	Control	37.23 (1.55)	32.35 (1.57)	29.27 (1.49)	*p*_g_ = 0.17
T1–T2	TCC	35.75 (1.76)	35.55 (1.69)	34.81 (1.52)	*p*_h_ = 0.24
	Control	39.61 (1.64)	37.20 (1.56)	36.6 (1.41)	*p*_g_ = 0.04*
T2–T3	TCC	24.22 (1.83)	20.12 (2.06)	17.56 (1.63)	*p*_h_ = 0.84
	Control	30.25 (1.69)	20.07 (1.92)	20.23 (1.52)	*p*_g_ = 0.02*
T3–T4	TCC	− 94.56 (7.11)	− 80.89 (5.53)	− 80.99 (6.67)	*p*_h_ = 0.10
	Control	− 105.67 (6.60)	− 97.98 (5.13)	− 89.42 (6.19)	*p*_g_ = 0.03*
T4–T5	TCC	18.83 (4.49)	16.92 (3.10)	15.73 (3.63)	*p*_h_ = 0.09
	Control	24.04 (3.97)	27.33 (2.73)	25.03 (3.20)	*p*_g_ = 0.04*
Peak	TCC	− 166.67 (16.40)	− 159.52 (10.80)	− 149.64 (11.80)	*p*_h_ = 0.74
	Control	− 222.41 (15.23)	− 191.10 (10.03)	− 169.81 (10.96)	*p*_g_ < 0.01*

*LL* leg length, *p*_*h*_ *p*-value for obstacle height, *p*_*g*_ *p*-value for group.

*Significant difference between subject groups.

**Figure 3:**
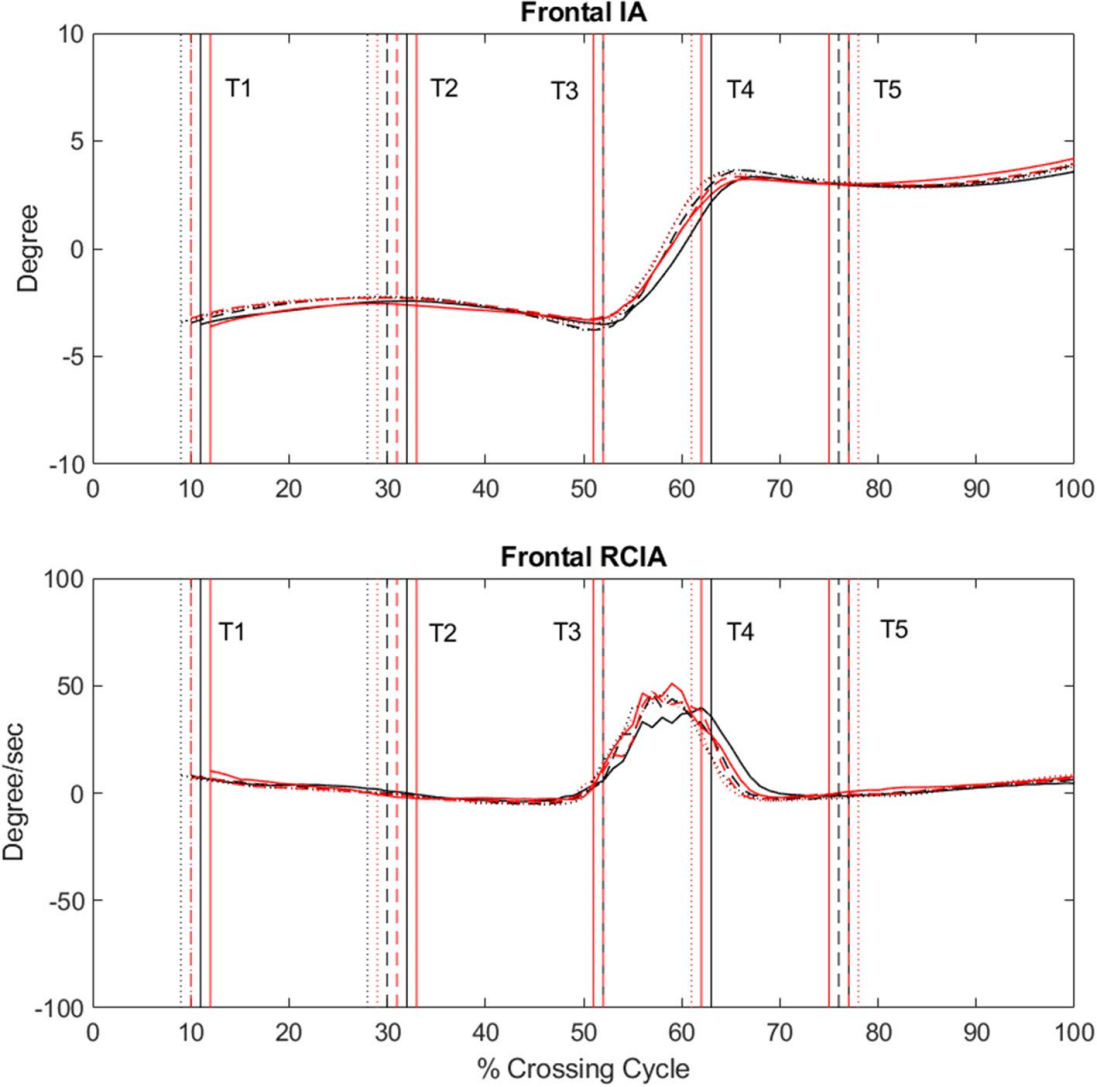
Ensemble-averaged frontal IA and RCIA for the TCC (black curves) and Control (red curves) groups when crossing obstacles of 10% LL (solid), 20% LL (dashed), and 30% LL (dotted). Vertical lines indicate selected crossing events: leading toe-off (T1); leading toe above the obstacle (T2); leading heel-strike (T3); trailing toe-off (T4); trailing toe above the obstacle (T5).

In the frontal plane, similar patterns in the IA and RCIA curves were found in both Control and the TCC group (Fig. 3). Quantitatively, between-group differences were found mainly in RCIA (Table 3). No significant between-group differences in IA were found. When compared to Control, the TCC group showed significantly smaller RCIA at leading-toe crossing (T2) and peak values during DLS, but significantly greater RCIA at trailing-toe crossing (T5) (Table 3). No significant height effects were found for any IA and RCIA variables in the frontal plane (Table 3).

**Table 3:** Adjusted means (standard errors, SE) of the frontal IA and RCIA at leading- and trailing-toe crossing, and of the average values over crossing sub-phases in the TCC and Control groups during obstacle-crossing. Also shown are results of a mixed-model two-way analysis of covariance (ANCOVA) with crossing speed as a covariate. (T1: leading toe-off; T2: leading toe above the obstacle; T3: leading heel-strike; T4: trailing toe-off; T5: trailing toe above the obstacle.)

Frontal plane	Group	Obstacle height (%LL)			Effects
		10	20	30	
**IA (degree)**					
T2	TCC	− 2.44 (0.18)	− 2.31 (0.16)	− 2.24 (0.15)	*p*_h_ = 0.78
	Control	− 2.64 (0.17)	− 2.27 (0.15)	− 2.28 (0.14)	*p*_g_ = 0.75
T5	TCC	2.96 (0.19)	3.08 (0.16)	3.07 (0.14)	*p*_h_ = 0.81
	Control	2.94 (0.18)	3.00 (0.15)	2.84 (0.13)	*p*_g_ = 0.62
T1–T2	TCC	− 2.79 (0.16)	− 2.60 (0.15)	− 2.61 (0.16)	*p*_h_ = 0.76
	Control	− 2.79 (0.15)	− 2.50 (0.14)	− 2.52 (0.15)	*p*_g_ = 0.74
T2–T3	TCC	− 2.97 (0.20)	− 2.93 (0.18)	− 2.84 (0.18)	*p*_h_ = 0.48
	Control	− 2.96 (0.19)	− 2.77 (0.17)	− 2.82 (0.17)	*p*_g_ = 0.79
T3–T4	TCC	− 0.72 (0.20)	− 0.63 (0.23)	− 0.55 (0.24)	*p*_h_ = 0.85
	Control	− 0.65 (0.19)	− 0.35 (0.22)	− 0.47 (0.22)	*p*_g_ = 0.59
T4–T5	TCC	3.11 (0.18)	3.33 (0.17)	3.40 (0.16)	*p*_h_ = 0.52
	Control	3.03 (0.17)	3.11 (0.6)	3.14 (0.15)	*p*_g_ = 0.39
**RCIA (degree/s)**					
T2	TCC	− 1.18 (0.66)	− 0.60 (0.45)	0.83 (0.61)	*p*_h_ = 0.97
	Control	− 2.92 (0.61)	− 0.91 (0.42)	− 0.94 (0.56)	*p*_g_ = 0.03*
T5	TCC	− 1.31 (0.47)	− 1.54 (0.51)	− 2.12 (0.43)	*p*_h_ = 0.65
	Control	0.56 (0.44)	− 0.77 (0.47)	− 1.33 (0.40)	*p*_g_ = 0.03*
T1–T2	TCC	2.87 (0.38)	2.91 (0.44)	3.01 (0.50)	*p*_h_ = 0.15
	Control	2.62 (0.35)	2.38 (0.41)	2.71 (0.46)	*p*_g_ = 0.48
T2–T3	TCC	− 1.14 (0.62)	− 2.43 (0.72)	− 2.30 (0.42)	*p*_h_ = 0.87
	Control	− 1.58 (0.56)	− 1.23 (0.67)	− 1.85 (0.40)	*p*_g_ = 0.51
T3–T4	TCC	30.60 (3.07)	31.81 (2.56)	34.76 (2.85)	*p*_h_ = 0.43
	Control	34.43 (2.85)	34.24 (2.40)	33.3 (2.64)	*p*_g_ = 0.63
T4–T5	TCC	4.45 (1.05)	2.50 (0.90)	1.50 (0.99)	*p*_h_ = 0.48
	Control	4.01 (0.98)	1.67 (0.84)	1.01 (0.92)	*p*_g_ = 0.61
Peak	TCC	48.89 (4.70)	48.35 (4.06)	49.92 (3.33)	*p*_h_ = 0.13
	Control	66.71 (4.36)	67.10 (3.77)	60.56 (3.08)	*p*_g_ < 0.01*

*LL* leg length, *p*_*h*_ *p*-value for obstacle height, *p*_*g*_ *p*-value for group.

*Significant difference between subject groups.

## Discussion

The current study aimed to determine the effects of long-term TCC practice and obstacle height on foot-clearances and whole-body balance control during obstacle-crossing in older people, in terms of IA and RCIA of the COM relative to COP. The TCC group was found to cross obstacles with significantly greater leading and trailing toe-obstacle clearances and significantly more posterior COM position relative to the COP throughout the crossing cycle, showing specific COM-COP control. The current results suggest that long-term TCC practitioners crossed obstacles with a more conservative COM-COP control strategy for less risk of tripping and better balance control compared to non-practitioners of TCC.

The TCC group was found to cross obstacles of different heights with significantly greater leading and trailing toe-obstacle clearances than controls without TCC experience. Previous studies have shown that increased clearance helps reduce the risk of tripping over obstacles^6,37^. Accompanied with the greater toe-obstacle clearance, the TCC group adopted a specific balance control strategy that involved a significantly more posterior COM position relative to the COP throughout the crossing cycle, showing greater posterior IA during the first half of leading-limb crossing (T1–T2), smaller anterior IA during the second half of leading-limb crossing (T2–T3) and DLS (T3–T4), and greater posterior IA during trailing-limb crossing (T4–T5) (Fig. 2). They also showed slower anterior COM movement relative to the COP as indicated by smaller sagittal RCIA magnitudes throughout the crossing cycle, except during DLS (T3–T4) showing smaller posterior RCIA and at leading-toe crossing (T2) and trailing-toe crossing (T5) showing greater frontal RCIA towards stance limb instead (Figs. 2 and 3). When the leading toe was above the obstacle (T2), the anterior IA was already very small for all obstacle heights in older people. However, young adults were able to reduce the anterior IA further to reach a COM position nearly right above the position of the COP in the sagittal plane for better stability at leading-toe crossing^5^. Significantly reduced anterior IA at T2 was also found in the TCC group when compared to the older non-TCC controls, suggesting that the body posture changes in the TCC group not only led to an increased leading toe-obstacle clearance but also a COM-COP position for better stability in the sagittal plane.

When the trailing toe was above the obstacle (T5) with greater toe-obstacle clearance, the TCC group kept the position of the body’s COM more posteriorly relative to the COP, which effectively increased the posterior IA. However, the anterior RCIA did not increase correspondingly, which would require greater joint torques in the stance limb to provide support to the body for the smooth COM-COP movement, as well as for precise control of the swing foot^14,19,20^. The current results indicate that the older long-term TCC practitioners had the necessary muscle strength to maintain stability during the trailing-foot crossing without the need to increase the corresponding RCIA.

During the DLS phase between the leading heel-strike and trailing toe-off (T3–T4), the COM was controlled within a relatively small range while the COP travelled from the trailing to the leading limb for the bodyweight transfer^42^. During this period, the older TCC practitioners also kept the position of the body’s COM more posteriorly relative to the COP with smaller average anterior IA and smaller posterior RCIA, suggesting a well-controlled, more conservative COM-COP motion for a smoother bodyweight transfer while maintaining dynamic balance^14,48^. Note that these observed COM-COP control features in the older TCC practitioners compared to non-TCC controls were not affected by obstacle height as both groups maintained the same crossing speed when crossing obstacles of increasing height. The smooth and stable transfer of the body weight over DLS during obstacle-crossing, the more posterior COM-COP in T2 and T5, and increased foot clearance suggest long-term TCC practitioners have better dynamic balance control during obstacle crossing than non-practitioners, in addition to a reduced risk of tripping.

The observed COM-COP control strategies in the TCC group who kept the position of the body’s COM more posteriorly relative to the COP during obstacle-crossing appeared to correspond to the main features of TCC basic movements, which emphasize maintaining the trunk in the upright position with the COM at a more posterior position when moving forward^49^. It has been reported that the sum of the percentage duration of fixing, forward, and backward movements was about 60%, and that of single-limb support was almost 70% in TCC training^50^. These movements emphasize maintaining balance during single-leg support and keeping the body weight more on the trailing limb during the slow weight-shifting of double-limb support, helping increase the muscle strength in the lower extremities and improving whole-body balance^25,29,30^. The current findings in the long-term TCC practitioners suggest that they had an improved ability for the dynamic balance and endpoint (foot) control during obstacle-crossing, most likely related to the training effects of TCC movements.

The current study was the first to document the effects of long-term TCC practice on foot-clearances and the whole-body balance control in terms of IA and RCIA in older people when crossing obstacles of different heights during walking. Although previous studies have reported the positive benefits of TCC training, including increased foot-obstacle clearance during obstacle crossing^36^ and improved standing and walking balance^25,33^, no studies have provided quantitative descriptions of the dynamic control (COM relative to the COP) during each phase of the crossing cycle to reveal the general control strategy employed by experienced older TCC practitioners, nor has the current literature reported on such COM-COP controls associated with increased leading and trailing toe-obstacle clearances. Nonetheless, the current study was limited to the dynamic balance control when negotiating fixed obstacles in healthy older adults with long-term TCC experience. It is also worth investigating the benefits of TCC in older people when negotiating unexpected obstacles that are commonplace in real-life conditions and are more challenging to cross. Further studies will be needed to determine the efficacy in older adults with compromised neuromusculoskeletal function, such as frailty, neurological diseases, and/or joint degeneration. Future longitudinal studies will be needed for determining direct correlations between muscle strength and the observed COM-COP control during obstacle-crossing in TCC practitioners. Studies on the joint kinematics and kinetics of the locomotor system associated with the observed COM-COP control strategies adopted by the TCC subjects will be needed to reveal the underlying mechanisms.

## Conclusions

Long-term TCC practitioners displayed an obstacle-crossing technique with a lesser risk of tripping and better balance control, as indicated respectively by significantly increased toe-obstacle clearances and a more posterior COM position relative to the COP with smaller IA and RCIA during leading-limb crossing, and greater posterior IA and frontal RCIA during trailing-limb crossing compared to non-practitioners of TCC. The observed between-group differences in the COM-COP control appeared to be related to the main features of TCC movements that emphasize maintaining the trunk in the upright position with the COM at a more posterior position when moving forward and keeping the body weight more on the trailing limb during the slow weight-shifting of double-limb support.
